# Pleasure and distress in health professors’ work practice

**DOI:** 10.1590/0034-7167-2025-0366

**Published:** 2026-05-08

**Authors:** Ariane da Silva Pires, Eugenio Fuentes Pérez, Francisco Gleidson de Azevedo Gonçalves, Helena Ferraz Gomes, Carlos Eduardo Peres Sampaio, Daniel da Silva Granadeiro, Tallita Mello Delphino, Norma Valéria Dantas de Oliveira Souza

**Affiliations:** I Universidade do Estado do Rio de Janeiro. Rio de Janeiro, Rio de Janeiro, Brazil

**Keywords:** Faculty, Universities, Work, Occupational Health, Health Personnel., Docentes, Universidades, Trabajo, Salud Laboral, Personal de Salud.

## Abstract

**Objectives:**

to analyze situations that generate pleasure and/or distress in teaching.

**Methods:**

this is a qualitative, descriptive, and exploratory study conducted at a public university in the state of Rio de Janeiro. Thirty faculty members from five health programs at the Biomedical Center participated. The data were analyzed using thematic content analysis, resulting in two categories.

**Results:**

the most prominent pleasure-generating circumstances were: interpersonal interactions with students and coworkers; the relevance of training as a means of personal and social change; and knowledge production. In contrast, issues related to distress refer to: a lack of public investment in education; overload due to a high workload; competitiveness among peers; insufficient infrastructure; and inadequate working conditions.

**Conclusions:**

experiences of pleasure and distress have both positive and negative impacts on professors’ psychophysical well-being, potentially promoting health or leading to illness.

## RESUMEN

**Objetivos:**

analizar situaciones que generan placer y/o sufrimiento en la docencia.

**Métodos:**

estudio cualitativo, descriptivo y exploratorio, realizado en una universidad pública del estado de Río de Janeiro. Participaron 30 profesores de cinco programas de salud del Centro Biomédico. Los datos se analizaron mediante análisis de contenido temático, lo que resultó en dos categorías.

**Resultados:**

las circunstancias generadoras de placer más destacadas fueron: las interacciones interpersonales con estudiantes y compañeros de trabajo; la relevancia de la formación como medio de cambio personal y social; y la producción de conocimiento. En contraste, los problemas relacionados con el sufrimiento se refieren a: falta de inversión pública en educación; sobrecarga laboral; competitividad entre pares; infraestructura insuficiente; y condiciones laborales inadecuadas.

**Conclusiones:**

las experiencias de placer y sufrimiento tienen impactos tanto positivos como negativos en el bienestar psicofísico del profesorado, pudiendo favorecer la salud o provocar enfermedades.

## INTRODUCTION

The work environment of a professor in higher education is complex and multifaceted, as it involves demands and discussions of a technical, scientific, social, political and economic nature^(1)^. Furthermore, higher education encompasses specific aspects, such as the three pillars of teaching, research, and outreach. These characteristics mobilize diverse and interconnected knowledge, which influences social reality^(2)^.

However, like any work activity, teaching can also generate pleasure and/or distress, since the world of work is constituted in a dialectical way, crossed by contradictions that impact daily professional life^(3,4)^. To analyze this dynamic, we considered it pertinent to adopt the constructs of Psychodynamics of Work, which focus on understanding the relationship between subject and activity, revealing how organizational demands are internalized and reinterpreted in subjective experience. This framework allows us to problematize how factors such as workload, recognition, autonomy, and interpersonal relationships intertwine with subjective dimensions, producing both fulfillment and burnout^(5)^.

The contemporary work environment, strongly influenced by the neoliberal model, has become a progressively more hostile space for workers, resulting in increasing levels of distress over satisfaction^(6)^. In Brazil, this ideology began to be systematically implemented in the 1990s, in line with policies of economic liberalization, privatization, and the flexibilization of labor relations. Anchored in the logic of competitiveness and maximizing productivity, neoliberalism promoted profound transformations in the world of work, expressed in the intensification of activities, outsourcing, the precariousness of employment relationships, and the reduction of social and labor rights. At the same time, the rhetoric of efficiency and meritocracy shifts responsibility for their success or failure onto the individual, obscuring the structural determinants that impact their autonomy and health^(7)^.

In teaching and healthcare, these effects become even more evident: multiple workdays, increasing demands for qualifications, low recognition, and a lack of adequate conditions are concrete manifestations of neoliberal rationality in historically overburdened professions. In this context, understanding workplace distress in light of this model allows us to articulate objective and subjective dimensions, revealing how neoliberalism exacerbates inequalities, increases pressure for results, and weakens collective mechanisms of resistance^(8)^.

Thus, this work configuration affects professors’ work, leading to low wages, unsatisfactory structure of educational institutions, hierarchization and bureaucratization, limited number of personnel and material resources, little recognition, accelerated work pace, versatility, and multifunctionality^(4)^.

Thus, work organizations can be potentially unhealthy, demanding the most from workers and providing the least, resulting in distress and work-related illness. Recognition is the transition point between the sequence: distress - work - reward - pleasure. Thus, appreciation can serve as a tool to transform distress at work into pleasure^(9)^.

Considering the complexity of professors’ work activities and the increasingly adverse context of the world of work, the following research question was selected: which situations in teaching work generate pleasure and/or distress?

The study’s relevance lies in fostering a timely discussion about the neoliberal model implemented in professors’ workplaces and its impacts on their well-being. It is also important for encouraging professors to adopt a critical stance regarding the current state of the teaching workforce. From this perspective, it may contribute to the development of strategies to mitigate or neutralize the negative impacts of work on professors’ health.

## OBJECTIVES

To analyze situations that generate pleasure and/or distress in teaching work.

## METHODS

### Ethical aspects

The study was conducted in accordance with national and international ethical guidelines and approved by the institution’s Research Ethics Committee, whose opinion is attached to this submission. In accordance with research ethics standards, the right to confidentiality and anonymity, as well as the right to withdraw from the study at any time, were guaranteed. After participants agreed to participate in the study, two copies of an Informed Consent Form were made available for them to sign: one copy for the participant and one for the researcher.

### Study design and theoretical-methodological framework

This is an exploratory, descriptive study with a qualitative approach, anchored in the theoretical framework of Christophe Dejours’ Psychodynamics of Work^(10)^. It was written according to the international guide Consolidated Criteria for Reporting Qualitative Research^(11)^.

### Study setting

The study was conducted at a public university located in the city of Rio de Janeiro, in southeastern Brazil. The university offers 32 undergraduate programs, which include various specializations, bachelor’s degrees, and undergraduate degrees. It has 46 *stricto sensu* graduate programs, offering students 42 academic master’s degrees, 23 doctoral degrees, and two professional master’s degrees, in addition to approximately 100 *lato sensu* graduate programs in various fields. It is part of the Rio de Janeiro Distance Higher Education Center, which also includes five other universities. The research setting was specifically the university’s Biomedical Center, which encompasses programs in biology, nursing, medicine, nutrition, and dentistry.

### Methodological procedures

Data were collected from September 2023 to March 2024 through semi-structured interviews guided by a script developed for this study. The first part of the instrument contained information about participants’ sociodemographic and work characteristics. The second part contained open-ended questions about situations that generate pleasure and distress related to their work as a professor.

Faculty members from undergraduate programs in biology, nursing, nutrition, medicine, and dentistry who had been employed by the institution for more than a year were included. This timeframe was considered because it was considered sufficient for them to discuss their experiences in the workplace in greater depth. This criterion was based on studies of Psychodynamics of Work^(10)^, which assert that workers need at least one year to understand the dynamics of the organization and the work process, in order to make their perceptions clearer and more in-depth. Professors on leave, on vacation, or assigned to other institutions during the data collection period were excluded.

Two techniques were used to recruit participants: I) convenience selection, which begins with a convenience sample (also called a voluntary sample), used to select the first five participants in this study (one from each course mentioned above); and II) snowball sampling, which allowed the first study participants to indicate other participants for the research^(12)^.

### Data source

Thirty professors participated, six from each program at the center (biology, nursing, medicine, nutrition, and dentistry). The rationale for this planning was to ensure a balance of data collected across all the programs investigated, thus enabling equal participation of faculty members per program.

Faculty members were approached in person at the teaching units that make up the institution’s Biomedical Center (nursing, medical, and dental schools, and biology and nutrition institutes), and were invited to voluntarily participate in the study. Interviews were scheduled in advance according to participants’ availability and took place individually, in person, in a private setting chosen by the participants or scheduled by the researcher at the faculty member’s teaching unit. The main location used for this purpose was the department’s office. All interviews were audio-recorded and transcribed verbatim by the lead researcher into a Microsoft Word^®^ document, adhering to the criteria for accurate transcription and transcreation. They lasted an average of 20 minutes. Data collection was conducted by a doctoral professor and a doctoral student trained in the technique.

### Data analysis

Data were processed using thematic content analysis, which requires distinct stages^(13)^: pre-analysis; material exploration or coding; treatment, inference, and interpretation of results. In the pre-analysis stage, skimming and exhaustive rereading of the material were performed to organize the data and achieve the corpus for analysis. In the material exploration or coding stage, 118 recording units were identified, subsequently grouped into two preliminary categories based on thematic similarities and differences. In the treatment, inference, and interpretation of results stage, inferences and interpretation of the content were made.

## RESULTS

It can be seen that, of the 30 professors, 26.6% (n=08) are between 33 and 40 years old or between 51 and 55 years old, followed by 20% (n=06) who are over 60 years old. There is also a predominance of women in teaching, with 56.6% (n=17), thus evidencing 43.4% (n=13) of male professors. In relation to marital status, 53.3% (n=16) were married, and 36.6% (n=11) were single.

When asked about their professional category, 20% of participants reported being biologists, nurses, physicians, nutritionists, and dentists. Regarding the time since graduation, the majority of participants (f=36.6%; n=11) graduated between 21 and 30 years of age or between 31 and 35 years of age (f=20%; n=06). Regarding the position held in the institution, there was a predominance of Adjunct Professor (f=50%; n=15), with a prevalence of over 30 years of teaching experience (f=30%; n=09), followed by those with 16 to 20 years of teaching experience (f=26.6%; n=08). Among the courses of study in which they worked as professors, participants cited biology, physical education, nursing, medicine, nutrition, dentistry, and psychology, with nursing and nutrition being the most frequently taught (f=40%; n=12). In relation to the type of qualification of professors, the majority (f=76.6%; n=23) held a doctoral degree.

Concerning weekly workload, the majority of the professors surveyed (f=96.7%; n=29) work 40 hours, with 70% (n=21) working full-time. As for whether or not professors had another employment relationship, those who did not work at another institution prevailed (f=83.4%; n=25), but 16.6% (n=05) had an external relationship. Among the places of work, the following stand out: the municipality, the state, the private education system (college), and private practice.

The final aspect to be addressed concerns participant socioeconomic characteristics. Of those interviewed, 83.4% (n=22) reported receiving more than ten minimum wages and claimed to be the primary breadwinners of their families (f=70%), having one (f=30%; n=09) to two (f=36.6%; n=11) dependents. Regarding participants’ views on their pay, professors’ opinions were mixed: 46.6% (n=14) considered their pay poor; 6.6% (n=02) considered their pay terrible; 40% (n=12) considered their pay good; and 6.6% (n=02) considered their pay indifferent.

Regarding research productivity grants, the majority of faculty reported not receiving any type of grant (90%; n=27), and only 10% (n=03) received this type of benefit. Among the three faculty members who received productivity grants, two were university pro-scientists and held other research incentive grants from the Brazilian National Council for Scientific and Technological Development and the *Fundação de Amparo à Pesquisa do Estado do Rio de Janeiro*.

After applying the thematic content analysis technique, 45 units of meaning (UMs)/topics emerged, which were grouped into two thematic categories named below: i) Situations that generate pleasure in the teaching work context; and ii) Situations that generate distress and difficulties in daily work.

### Situations that generate pleasure in the teaching work context

This first category corresponded to 19 UMs, equivalent to 16.10% of the total number of recording units in the study. This category contains content representative of situations that generate pleasure.

The feeling of pleasure emerges from an appreciation for teaching as a profession, as working in teaching, including clinical teaching, with research development, especially supervising scientific work and university outreach activities, was highly emphasized as a source of great satisfaction.

*I really enjoy being in the classroom, guiding my students, whether they are monitors, undergraduate students, or those doing their final project, as well as master’s and doctoral students.* (I10 - faculty/nutrition)

*Well, situations of pleasure, for sure, is the activity of teaching, especially bedside teaching.* (I26 - faculty/medicine)

*Pleasure is more closely associated with practices related to outreach activities. Outreach activities give me much more freedom to create, to develop work from the perspective of autonomy and transformation.* (I2 - faculty/nursing)

In terms of interpersonal relationships, the pleasure that arises from the faculty-student relationship includes the establishment of positive connections, mutual respect, the exchange of information, and students’ ability to deal with the challenges of working in the public sector. Participants also demonstrated good relationships with their colleagues, which reflects teamwork and solidarity within the professional community.

*So, I think this contact with the students is very enjoyable, with a fantastic team of professors. So, they are very inspiring people for me. This is very enjoyable.* (I11 - faculty/nutrition)

*Working with students on both technical and day-to-day matters is a great pleasure. Also, interacting with colleagues, some longtime, others newer, are the highlights that bring pleasure.* (I19 - faculty/dentistry)

Another pleasurable aspect is the opportunity to monitor a student’s academic development in ethical and professional terms and to see that their education served as an important means of social and personal transformation. Professors feel rewarded by being an integral part of the process of building a professional future.

*It’s very rewarding to take on a student at an early stage and witness their growth in the development of this future professional. I see changes in both the personal and social aspects of citizenship. It’s a pleasure to witness this transformation.* (I6 - faculty/biology)

*The good part is when you can monitor your student’s academic growth and the development of their professional ethics. I realize that, over the years, I’ve been able to pass on, teach, at least part of my experience to my students.* (I3 - faculty/medicine)

Furthermore, they pointed out as a situation that generates pleasure an environment that offers the possibility of producing knowledge, which can be achieved through research and publishing articles.

*Being able to build knowledge, immortalize some type of knowledge through the material produced, whether by publishing an article, presenting it at an event, holding a conversation circle, or even having a discussion, is also a source of pleasure.* (I1 - faculty/dentistry)

*When we succeed with our work and get published, it’s great, like, for example, the launch of a book and its impact outside of Rio de Janeiro. So, completing this is very gratifying, because it’s the reward after such a huge effort in terms of energy.* (I9 - faculty/nutrition)

It was learned that circumstances that promote pleasure at work have a positive impact on health, as they increase feelings of satisfaction, belonging and recognition, in addition to strengthening professional identity and subjectivity.

*My work transforms me, and as it transforms me, it makes me stronger, reinforces my identity, and this has a positive impact on my body. So, I think in this sense, work rebuilds me and brings me good things from a health perspective.* (I24 - faculty/nursing)

*Work is good for my health because professionals who enjoy their work get a good return. My health is good precisely because I do the things that give me pleasure.* (I18 - faculty/medicine)

### Situations that generate distress and difficulties in daily work

This second category corresponded to 26 UMs, equivalent to 22% of the total number of recording units, which presented the content representing distress in and through work. Thus, professors stated that the lack of investment in science and technology and the budget cuts for public universities cause distress and hinder the development of teaching activities, as these resources are essential for professional life, as evidenced in participants’ statements.

*The distress is related to research, to the issue of funding, and the cost of developing the research. So, the dissatisfaction is not being able to move forward, to take longer steps due to financial constraints, which are much more difficult.* (I13 - faculty/nutrition)

*The difficulties are the conditions we find ourselves in, which have been worsening in recent years. Their colony of rats can no longer reproduce because there’s no more room in the vivarium, because there’s no food, because the air conditioning broke. The non-payment of research grants. All of this has been a major hindrance.* (I15 - faculty/biology)

Professors emphasize that work ends up encroaching on non-work time due to the diversity and complexity of tasks, and the high labor demands. This occurs because they are unable to complete all activities during formal work hours, which forces them to take home tasks they were unable to complete at work. This situation is an example of the versatility and multifunctionality of teaching.

*I can categorically state that we all work more than 40 hours a week, and we start to naturalize the weekends as work hours for what we can’t handle during the week. This causes distress, because when I go to bed at 3 a.m., it’s a movie I didn’t go to, it’s time with my son I didn’t have*. (I11 - faculty/dentistry)

*In addition to teaching, we also provide care work, which is significant. The demand for this work is crucial to keeping the service running, so we also have to be a care workforce. It gives the impression that professors only care for students, when in fact we have a workload related to patients and service administration.* (I17 - faculty/medicine)

The competitiveness that exists in the university workplace was highlighted by participants as a source of distress. However, dialectically, in the previous category, good relationships with colleagues were mentioned as an aspect that constitutes pleasure.

*Sometimes we feel like giving up because we can’t compete with large centers and with the competition here among faculty. When we have “little to go”, the groups that hold power end up finding ways to maintain that power, which creates unfair or fierce competition and discourages us.* (I27 - faculty/biology)

*You can’t count on your coworker, because they seem to feel threatened in the presence of another. Another thing that needs to be said is that the academic/scientific world is a world of social division of labor and a very competitive world.* (I29 - faculty/nursing)

Inadequate working conditions, combined with the university’s precarious infrastructure, hinder professional growth, both in terms of physical infrastructure and in terms of material resources necessary for teaching practice in teaching and/or assistance, which have been associated with the situation of distress.

*Working conditions are poor, and the availability of physical and structural resources is fragile, almost nonexistent. My administrative support is poor, as is the equipment; the IT equipment is obsolete.* (I2 - faculty/nursing)

*There are physical limitations, such as within the medical school and within the hospital. There are very few areas that can be used for teaching, that is, bringing students together to discuss cases. In short, you don’t have a classroom; there are physical limitations and limited equipment for teaching.* (I3 - faculty/medicine)

*Another very important issue is the unhealthiness, because they don’t consider it unhealthy here, but there’s the sewage from the clinic upstairs: saliva, blood, because there’s surgery. These are usually infected surgeries, to remove abscesses, that kind of thing. So, we breathe that in because we’re closed and have air conditioning, so you’re exposed to it*. (I7 - faculty/dentistry)

## DISCUSSION

The results revealed a variety of situations that cause pleasure and distress experienced by professors. Feelings of satisfaction and dissatisfaction, stimulation and discouragement, well-being, and restlessness were identified, all of which are not neutral in relation to workers’ health, and can therefore either ensure or destabilize their health^(1)^.

It has been demonstrated that professors who are satisfied with their work can contribute to a dynamic, critically reflective, and high-quality training process. Satisfied and fulfilled professionals demonstrate a commitment to teaching, research, and outreach and are willing to systematically help students learn. Furthermore, research conducted in southern Brazil emphasized that happy professors have the ability to create new knowledge, share knowledge, integrate teaching with assistance and service, and develop citizens better prepared for the dynamic reality of modern society^(14)^.

Thus, teaching, research, and extension integration enhances academic work, brings the university closer to society, and enhances critical thinking. It also highlights the social side of academic practice^(15)^. When it comes to interpersonal relationships between individuals, communication enables the sharing of knowledge, as well as feelings, emotions, and perspectives. Good interpersonal relationships foster interaction between people and strengthen workplace bonds, making the environment more pleasant and conducive to task completion. With a network of friends, people feel safe and welcomed, developing skills to overcome obstacles, which can extend their employment tenure^(16)^.

From this perspective, social interactions between faculty and students enable the creation of cordial and/or friendly bonds in the workplace and are beneficial for both the employees’ health and the workplace’s organizational structure. Thus, when workplace relationships are beneficial, demonstrating respect, listening, interaction, and cooperation, pleasure prevails, and employees’ individuality is respected, in addition to the professional group’s health and well-being^(17)^.

The Psychodynamics of Work emphasizes that the capacity for change, creativity, collaboration, trust, and the perception of social usefulness are crucial elements for the feeling of pleasure in work and in the work itself^(18)^.

In the field of study, affinity for a specific subject and the exchange of thoughts and reflections through debates and scientific research are known to aid in the socialization process. This interaction between professor and student is extremely beneficial both for professors, who have the opportunity to feel heard and appreciated in their field of study, and for students, who can gain knowledge from someone with extensive experience in the subject^(19)^.

A systematic review highlighted that academic careers have enabled the connection between teaching and research, in addition to intensifying the training of new researchers and technical publications. This represents an opportunity for self-fulfillment, as it is an activity seen as highly^(20)^.

Therefore, it is the professors’ responsibility to address topics related to the principles of the profession and the advanced knowledge emerging from recent studies with students. Furthermore, it is highly recommended that professors produce knowledge through research, making groundbreaking discoveries and disseminating them through the publication of scientific articles in high-impact international journals^(20)^.

In higher education institutions, knowledge generation is intrinsic to professors’ work. These professionals are assessed both quantitatively and qualitatively, which encourages them to remain productive in academic environments, leading to greater prominence in their teaching careers^(21)^.

The results in the second category highlight that globalization, economic trends, and competitiveness in teaching have led to changes in work organization, demanding accelerated professional performance. This results in transformations in public and private companies that prioritize the use of high technology, nanotechnology, artificial intelligence, and other technologies. This context demands rapid adaptation, directly impacting professors’ physical and mental health. This has led to increased stress rates among workers, and university teaching is not immune to this situation^(22)^.

The lack of investment in the public higher education sector was evident in participants’ statements, as maintenance and investment in basic equipment, such as computers, air conditioning units, and others, are inadequate, causing distress. Added to this are budget cuts that hinder teaching, and without financial incentives, many research projects are rendered unviable^(23)^.

Overload and accumulation of tasks force professors to adapt to a fast-paced work schedule. This makes it difficult for them to mentally disconnect from their responsibilities and feel guilty when taking a break, thinking about pending tasks. Many become frustrated because they only feel useful when they are working intensely^(24,25)^.

This result is similar to that of a study conducted with professors from a public institution located in all regions of Brazil, which revealed dissatisfaction with the work structure due to pressure to meet deadlines, increased time dedicated to work, demand for results and increased workload for professors^(26-28)^.

In this way, professors end up performing tasks in domestic environments, in their homes, including supervising research and final projects, writing scientific articles, filling out forms for funding entities, carrying out assessments for scientific journals, among other tasks^(19)^.

It is important to highlight that this invasion of personal space by the corporate environment can negatively affect professors not only because they have to sacrifice their rest and leisure time to fulfill their work obligations, but also because these professionals need to use their own material resources, such as computers, internet, electricity, among others, also affecting their financial expenses^(29)^.

Hence, the overload related to the needs and excess workload present in research, teaching, and extension resulting from accumulation of work among faculty, considering the disproportionate student-faculty ratio, as well as the variety of tasks in undergraduate, graduate, research, and extension programs, are detrimental to faculty well-being. These activities, along with administrative and bureaucratic tasks, are intensified by the shortage of technical and administrative staff^(27)^.

This circumstance is related to the high pressure for productivity imposed by targets, a hallmark of the neoliberal context. In this scenario, professors’ excessive workload stands out. Teaching is now marked by the weakening of interpersonal bonds and professional relationships, in addition to accumulation of responsibilities that intensify the pace of work in educational institutions. This creates a new work structure in education, which favors individual assessment methods, encourages competition, and moves away from science as a collective practice, prioritizing the number of academic publications^(25)^.

These results point to the particularities and demands of the work process of master’s and doctoral professors linked to workaholism. Thus, workaholics work excessively and compulsively, sacrificing leisure time or social and family interaction for the sake of intense work. However, they often fail to achieve the expected performance, as workaholism increases the likelihood of work incapacity due to deterioration in biopsychosocial health^(30)^.

Competitiveness and vanity are intrinsic characteristics of academic environments, as it is home to individuals of high social standing, holders and disseminators of advanced scientific knowledge. However, it is noteworthy that, with the implementation of the neoliberal model, this situation has intensified^(31)^.

It is also worth highlighting that the appropriation of workers’ subjectivity is linked to the characteristics of neoliberal capitalism, where workers’ subjectivity is severely impacted by competition and individualism promotion and encouragement, generated by the restructuring of labor relations^(32)^.

Evidence suggests that neoliberalism employs tactics that expand control over workers’ subjectivity, aiming to foster fierce competition and individualism. This increases disunity among workers and their organizational abilities, resulting in a hostile work environment in terms of interpersonal relationships^(33)^.

It is worth noting that, at the beginning of the 21^st^ century, with the accelerated growth of higher education institutions in Brazil, the Coordination for the Improvement of Higher Education Personnel (In Portuguese, *Coordenação de Aperfeiçoamento de Pessoal de Nível Superior* - CAPES) began to implement some assessment criteria to categorize these institutions in the areas of research, teaching, and extension. The fundamental assessment criteria include the work schedule of most faculty members, their research competence, the structure for producing and disseminating scientific knowledge, and *stricto sensu* graduate programs. In other words, for an institution to achieve a good CAPES rating, it needs a highly qualified faculty, fully dedicated to the university, committed to producing and disseminating high-quality and quantitative research, which results in fundraising for the institution and its faculty^(27)^.

Thus, in addition to the responsibilities inherent to the teaching profession, the teaching environment has fostered a relentless pursuit of productivity, evidenced by publications resulting from studies in internationally renowned journals. Therefore, research incentive agencies and universities encourage faculty to produce large-scale scientific articles, which results in greater career recognition, increased visibility for the university, and financial benefits through research grants, career advancement, and institutional funding for further research^(34)^.

Inadequate infrastructure was also addressed by professors, resulting from the deterioration of working conditions, marked by poorly planned spaces, lack of rooms for workers, insufficient and/or inadequate lighting, among other aspects, which has been highlighted as an obstacle to the advancement of work in the public sector.

Thus, the physical infrastructure of public educational and healthcare institutions is often inadequate, presenting a series of distortions such as: rooms of inadequate sizes for the target audience; narrow corridors; lack of ramps; poor lighting; insufficient ventilation or air conditioning; old and deteriorated physical facilities; insufficient bathrooms for the number of workers; scarcity of material resources; poor maintenance of existing resources; or even obsolete resources^(27)^.

This scarcity and inadequate working conditions cause profound psychological distress, illness, and even an increase in career abandonment rates. This work environment presents challenges for workers in performing their duties, and the tasks performed undoubtedly fall short of what is required^(30)^.

It should be noted that distress is seen as a subjective experience intermediate between uncontrolled mental illness and psychological comfort (or well-being). Therefore, the debate does not end with distress itself, but with the understanding that it can arise as a pathological condition. From this perspective, pathological distress arises when all opportunities for adaptation or adjustment to the organization of work by individuals have been exhausted and the subjective connection with this organization of work is impeded^(35)^.

Thus, when work reorganization becomes unfeasible, when the relationship between the worker and the organization becomes very disconnected, i.e., if the organization’s demands or working conditions are extremely adverse, workers are unable to establish a healthy process of adaptation through any type of mediation, resulting in pathological distress and illness^(36)^.

### Study limitations

A limitation of this study is that it was conducted in a single setting, which may compromise the generalizability of results. Therefore, we suggest conducting research in other public and private settings and other fields of knowledge, such as the humanities and exact sciences, to assess the specificities of areas other than healthcare.

### Contributions to nursing

The study contributes significantly to the field of occupational health and training, reinforcing the need for a balance between situations that generate pleasure and distress in university health education. These contributions can guide institutional actions, educational policies, and pedagogical practices committed to faculty health and favorable working conditions for excellence in health education at Brazilian public universities.

## FINAL CONSIDERATIONS

Concerning professors’ views on the circumstances that generate pleasure and distress at work, it was found that the circumstances that provide pleasure are linked to: the progress of teaching, research, and extension activities; interpersonal interactions with students and coworkers; the relevance of training as a means of mobilization and personal and social change; and the production of knowledge materialized in books, articles, and scientific works.

The circumstances identified as causing distress and challenges in teaching work were: the lack of public investment in education and, consequently, in universities; the overload of activities due to the large volume of work; the competition present in the university environment; insufficient infrastructure; and inadequate working conditions.

## Data Availability

The research data are available within the article.
