# Supplementary material for: Pleasure and distress in health professors’ work practice
Source: Rev Bras Enferm. 2026 May 8;79:e20250366. doi: 10.1590/0034-7167-2025-0366 (PMC13169629; doi:10.1590/0034-7167-2025-0366)
Supplement: Translation

# Translation: Prazer e sofrimento na prática laboral de docentes universitários na área da saúde

**DOI:** 10.1590/0034-7167-2025-0366pt

**Authors:** Ariane da Silva Pires, Eugenio Fuentes Pérez, Francisco Gleidson de Azevedo Gonçalves, Helena Ferraz Gomes, Carlos Eduardo Peres Sampaio, Daniel da Silva Granadeiro, Tallita Mello Delphino, Norma Valéria Dantas de Oliveira Souza

**Affiliations:** I Universidade do Estado do Rio de Janeiro. Rio de Janeiro, Rio de Janeiro, Brasil

**Keywords:** Docentes, Universidades, Trabalho, Saúde Ocupacional, Pessoal da Saúde.

## Abstract

**Objetivos:**

analisar as situações geradoras de prazer e/ou de sofrimento no trabalho docente.

**Métodos:**

trata-se de estudo qualitativo, descritivo e exploratório, realizado em uma universidade pública situada no estado do Rio de Janeiro. Participaram 30 docentes do Centro Biomédico de cinco cursos da saúde. Os dados foram examinados mediante a técnica de análise de conteúdo temática, emergindo duas categorias.

**Resultados:**

as circunstâncias geradoras de prazer destacadas foram: as interações interpessoais com os alunos e colegas de trabalho; a relevância da formação como meio de mudanças pessoais e sociais; e a produção de conhecimento. Em contrapartida, as questões relacionadas ao sofrimento referem-se à: escassez de investimentos públicos na educação; sobrecarga devido ao grande volume de trabalho; competitividade entre os pares; infraestrutura insuficiente; e condições de trabalho inadequadas.

**Conclusões:**

as experiências de prazer e sofrimento apresentam impactos positivos e negativos na dimensão psicofísica dos docentes, podendo assegurar saúde ou conduzir ao adoecimento.

## INTRODUÇÃO

O ambiente de trabalho do professor no ensino superior é complexo e multifacetado, pois envolve demandas e discussões de natureza técnica, científico, social, político e econômico^(1)^. Ademais, o trabalho na educação superior contempla especificidades, como o tripé ensino, pesquisa e extensão. Tais características mobilizam conhecimentos variados e interligados, os quais influenciam a realidade social^(2)^.

Porém, como toda atividade laboral, a docência também pode gerar prazer e/ou sofrimento, uma vez que o mundo do trabalho se constitui de forma dialética, atravessado por contradições que impactam o cotidiano profissional^(3,4)^. Para analisar essa dinâmica, considerou-se pertinente adotar os construtos da Psicodinâmica do Trabalho, que se dedicam a compreender a relação entre sujeito e atividade, revelando como as exigências organizacionais são internalizadas e ressignificadas na experiência subjetiva. Esse referencial permite problematizar como fatores como carga horária, reconhecimento, autonomia e relações interpessoais se entrelaçam às dimensões subjetivas, produzindo tanto realização quanto desgaste^(5)^.

O ambiente contemporâneo de trabalho, fortemente influenciado pelo modelo neoliberal, tem se configurado como espaço progressivamente mais hostil ao trabalhador, resultando em níveis crescentes de sofrimento em detrimento da satisfação^(6)^. No Brasil, esse ideário passou a ser implementado de forma sistemática na década de 1990, em sintonia com políticas de abertura econômica, privatizações e flexibilização das relações laborais. Ancorado na lógica da competitividade e da maximização da produtividade, o neoliberalismo promoveu transformações profundas no mundo do trabalho, expressas na intensificação das atividades, na terceirização, na precarização dos vínculos, e na redução de direitos sociais e trabalhistas. Ao mesmo tempo, a retórica da eficiência e da meritocracia desloca para o indivíduo a responsabilidade por seu sucesso ou fracasso, ocultando as determinações estruturais que impactam sua autonomia e saúde^(7)^.

Na docência e na saúde, esses efeitos se tornam ainda mais evidentes: múltiplas jornadas; crescente exigência por qualificação; baixo reconhecimento; e ausência de condições adequadas configuram manifestações concretas da racionalidade neoliberal sobre profissões historicamente sobrecarregadas. Nesse contexto, compreender o sofrimento laboral à luz desse modelo permite articular dimensões objetivas e subjetivas, revelando como o neoliberalismo acentua desigualdades, amplia a pressão por resultados e fragiliza mecanismos coletivos de resistência^(8)^.

Assim, esta configuração laboral afeta o trabalho dos docentes, levando a salários baixos, estrutura insatisfatória das instituições educacionais, hierarquização e burocratização, quantidade limitada de pessoal e recursos materiais, pouco reconhecimento, ritmo de trabalho acelerado, polivalência, e multifuncionalidade^(4)^.

Desse modo, as organizações laborais podem ser potencialmente adoecedoras, exigindo o máximo dos trabalhadores e proporcionando o mínimo, resultando em sofrimento e adoecimento pelo trabalho. O reconhecimento é o ponto de transição entre a sequência: sofrimento - trabalho - recompensa - prazer. Desse modo, a valorização pode funcionar como instrumento para transformar o sofrimento no trabalho em prazer^(9)^.

Considerando a complexidade da atividade laboral do professor e o contexto cada vez mais adverso do mundo do trabalho, selecionou-se como questão de pesquisa: quais as situações do trabalho docente são geradoras de prazer e/ou sofrimento?

A relevância do estudo se caracteriza em fomentar uma discussão oportuna sobre o modelo neoliberal implementado no ambiente de trabalho dos docentes e seus impactos para o bem-estar desses profissionais. Também tem importância por estimular uma postura crítica nos docentes em relação à atual situação do mundo laboral no campo do ensino. Sob essa ótica, poderá contribuir para elaboração de estratégias para atenuar ou neutralizar os impactos negativos do trabalho na saúde dos docentes.

## OBJETIVOS

Analisar as situações geradoras de prazer e/ou de sofrimento no trabalho docente.

## MÉTODOS

### Aspectos éticos

O estudo foi conduzido conforme diretrizes éticas nacionais e internacionais, aprovado pelo Comitê de Ética em Pesquisa da instituição, cujo parecer está anexado à presente submissão. Em conformidade com as normas éticas de pesquisa, garantiram-se o direito ao sigilo e anonimato, e o direito de desistir a qualquer momento do estudo. Após a aceitação em participar do estudo, foi disponibilizado um Termo de Consentimento Livre e Esclarecido em duas vias, para que cada participante assinasse: uma via para o próprio participante e outra para o pesquisador.

### Tipo de estudo e referencial teórico-metodológico

Pesquisa exploratória, descritiva, com abordagem qualitativa, ancorada no referencial teórico da Psicodinâmica do Trabalho de Christophe Dejours^(10)^. Foi redigida conforme o guia internacional *Consolidated Criteria for Reporting Qualitative Research* ^(11)^.

### Cenário do estudo

O estudo foi desenvolvido em uma universidade pública situada no município do Rio de Janeiro, no sudeste do Brasil. A referida universidade oferece 32 cursos de graduação, que se desdobram em diferentes habilitações, licenciaturas e bacharelados. Possui 46 programas de pós-graduação *stricto sensu*, proporcionando aos estudantes 42 cursos de mestrado acadêmico, 23 cursos de doutorado e dois cursos de mestrado profissional, além de aproximadamente 100 cursos de pós-graduação *lato sensu* em diversas áreas do conhecimento. Integra o Centro de Educação Superior a Distância do Rio de Janeiro, do qual também fazem parte outras cinco universidades. O cenário de investigação foi, especificamente, o Centro Biomédico dessa universidade, que engloba os cursos de biologia, enfermagem, medicina, nutrição e odontologia.

### Procedimentos metodológicos

Os dados foram coletados de setembro de 2023 a março de 2024, por meio de entrevista semiestruturada, guiada por um roteiro elaborado para este estudo. A primeira parte do instrumento continha informações acerca da caracterização sociodemográfica e laboral dos participantes. A segunda parte continha questões abertas sobre as situações geradoras de prazer e sofrimento relacionadas ao seu trabalho como docente universitário.

Docentes dos cursos de graduação em biologia, enfermagem, nutrição, medicina e odontologia, com vínculo empregatício estatutário e que estivessem em pleno exercício de suas funções há mais de um ano na instituição foram incluídos. Considerou-se esse recorte temporal de atuação profissional por entender que era suficiente para que pudessem discorrer sobre vivências no ambiente laboral com maior profundidade. Esse critério se fundamentou em estudos da Psicodinâmica do Trabalho^(10)^, os quais asseveram que o trabalhador necessita de, pelo menos, um ano para apropriar-se da dinâmica da organização e do processo de trabalho, a fim de tornar suas percepções mais claras e aprofundadas. Docentes licenciados, em período de férias ou cedidos a outras instituições no período de coleta de dados foram excluídos.

Foram utilizadas duas técnicas de captação de participantes: I) seleção por conveniência, quando se inicia com uma amostra de conveniência (também denominada amostra voluntária), utilizada para seleção dos cinco primeiros participantes deste estudo (um de cada curso mencionado anteriormente); e II) bola de neve, que permitiu que os primeiros participantes do estudo indicassem outros participantes para a pesquisa^(12)^.

### Fonte de dados

Participaram 30 docentes universitários, sendo seis de cada curso do referido centro (biologia, enfermagem, medicina, nutrição e odontologia). A justificativa para esse planejamento foi garantir o equilíbrio dos dados coletados entre todos os cursos investigados, podendo, dessa maneira, oportunizar uma participação equânime de docentes por curso.

Os docentes foram abordados de forma presencial nas unidades de ensino que compõem o Centro Biomédico da instituição (faculdades de enfermagem, medicina, odontologia e institutos de biologia e nutrição), sendo convidados a participar voluntariamente do estudo. As entrevistas foram agendadas previamente conforme disponibilidade dos participantes, e ocorreram de forma individual, presencial, em ambiente privativo, escolhido por eles ou agendado pelo pesquisador na própria unidade de ensino do docente, sendo o principal local utilizado para esta finalidade a sala do departamento. Todas as entrevistas foram gravadas em áudio e transcritas na íntegra pela pesquisadora principal em um documento de texto no *Microsoft Word* ^®^, respeitando os critérios de fidelidade da transcrição e transcriação. Tiveram duração média de 20 minutos. A coleta de dados foi conduzida por uma docente doutora e uma doutoranda devidamente capacitada na técnica.

### Análise dos dados

O tratamento dos dados ocorreu por meio da técnica de análise de conteúdo temática, que demanda etapas distintas^(13)^: pré-análise; exploração do material ou codificação; tratamento, inferência e interpretação de resultados. Na etapa de pré-análise, foram realizadas leitura flutuante e a releitura exaustiva do material, a fim de organizar os dados e alcançar o *corpus* de análise. Na etapa de exploração do material ou codificação, foram identificadas 118 unidades de registro, posteriormente agrupadas em duas categorias preliminares conforme semelhanças e diferenças temáticas. Na etapa de tratamento, inferência e interpretação de resultados, foram realizadas inferências e interpretação do conteúdo.

## RESULTADOS

Verifica-se que, dos 30 docentes, 26,6% (n=08) têm entre 33 e 40 anos ou entre 51 e 55 anos, seguidos de 20% (n=06) com mais de 60 anos. Constata-se também o predomínio feminino na docência, com 56,6% (n=17), evidenciando-se, assim, 43,4% (n=13) de professores do sexo masculino. Sobre o estado civil, 53,3% (n=16) eram casados, e 36,6% (n=11) eram solteiros.

Ao serem questionados sobre a categoria profissional, 20% dos participantes refeririam ser biólogos, enfermeiros, médicos, nutricionistas e odontólogos. Em relação ao tempo de conclusão da graduação, a maioria dos participantes (f=36,6%; n=11) estava formada entre 21 e 30 anos ou entre 31 e 35 anos (f=20%; n=06). No que concerne ao cargo ocupado na instituição, houve predomínio do Professor Adjunto (f=50%; n=15), prevalecendo o tempo de atuação na docência superior a 30 anos (f=30%; n=09), seguido daqueles com 16 a 20 anos de experiência docente (f=26,6%; n=08). Entre os cursos de atuação como docentes, os participantes citam biologia, educação física, enfermagem, medicina, nutrição, odontologia e psicologia, sendo os cursos de maior atuação a enfermagem e a nutrição (f=40%; n=12). Quanto ao tipo de qualificação dos professores, a maioria (f=76,6%; n=23) possuía o título de doutor.

Quanto à carga horária semanal, a maioria dos docentes investigados (f=96,7%; n=29) exerce 40 horas de atividades laborais, sendo 70% (n=21) sob o regime de dedicação exclusiva. A respeito dos docentes possuírem ou não outro vínculo empregatício, prevaleceram aqueles que não trabalhavam em outra instituição (f=83,4%; n=25), porém 16,6% (n=05) possuíam vínculo externo. Entre os locais de atuação, citam-se o município, o estado, a rede privada de ensino (faculdade) e o consultório particular.

O último aspecto a ser abordado se refere à caracterização socioeconômica dos participantes. Dos entrevistados, 83,4% (n=22) declararam receber mais de dez salários mínimos e afirmaram ser os principais mantenedores do núcleo familiar (f=70%), tendo de um (f=30%; n=09) a dois (f=36,6%; n=11) dependentes. Em relação ao ponto de vista dos participantes acerca de sua remuneração, a opinião dos docentes foi heterogênea: 46,6% (n=14) consideraram a remuneração ruim; 6,6% (n=02) consideraram a remuneração péssima; 40% (n=12) julgaram a remuneração boa; e 6,6% (n=02) julgaram a remuneração indiferente.

Em relação às bolsas de produtividade de pesquisa, prevaleceram os docentes que informaram não possuir nenhum tipo de bolsa (90%; n=27), e apenas 10% (n=03) tinham esse tipo de benefício. Entre os três docentes contemplados com bolsa de produtividade, dois eram procientistas da universidade e acumulavam outras bolsas de incentivo à pesquisa do Conselho Nacional de Desenvolvimento Científico e Tecnológico e Fundação de Amparo à Pesquisa do Estado do Rio de Janeiro.

Após aplicação da técnica de análise de conteúdo temática, emergiram 45 unidades de significação (USs)/temas, que foram agrupadas em duas categorias temáticas denominadas a seguir: i) Situações geradoras de prazer no contexto laboral docente; e ii) Situações geradoras de sofrimento e de dificuldades no cotidiano laboral.

### Situações geradoras de prazer no contexto laboral docente

Essa primeira categoria correspondeu a 19 USs, o que equivale a 16,10% do total de unidades de registro do estudo. Nessa categoria, encontram-se os conteúdos representativos de situações geradoras de prazer.

O sentimento de prazer emerge pelo apreço pela docência enquanto profissão, pois atuar no ensino, inclusive ensino clínico, com o desenvolvimento de pesquisas, destacando-se orientações de trabalhos científicos e atividades de extensão universitária, foi muito enfatizado como de grande satisfação.

*Eu gosto muito de estar em sala de aula, orientar meus alunos, sejam monitores, alunos de iniciação científica, ou que estejam fazendo trabalho de conclusão de curso, e também mestrandos e doutorandos.* (E10 - docente/nutrição)

*Bom, situações de prazer, com certeza, é a atividade de ensino, especialmente o ensino à beira do leito.* (E26 - docente/medicina)

*O prazer está mais associado às práticas relacionadas às atividades de extensão. A atividade de extensão me dá muito mais liberdade de criar, de desenvolver um trabalho a partir da perspectiva de autonomia de transformação.* (E2 - docente/enfermagem)

No âmbito das relações interpessoais, o prazer que surge da relação docente-discente inclui o estabelecimento de conexões positivas, o respeito mútuo, a troca de informações e a capacidade dos alunos de lidar com os desafios que envolvem o mundo do trabalho no setor público. Os participantes também demonstraram boas relações com os colegas, o que significa trabalho em equipe e solidariedade no coletivo profissional.

*Então, esse contato com os alunos eu acho que é muito prazeroso, com uma equipe de professores que é fantástica. Então, são pessoas muito inspiradoras para mim. Isso é muito prazeroso.* (E11 - docente/nutrição)

*A convivência com os alunos tanto nas questões técnicas como nas questões do dia a dia gera muito prazer. Também a convivência com colegas, alguns de longa data outros mais novos que estão entrando; são os pontos altos que dão prazer.* (E19 - docente/odontologia)

Outra situação prazerosa está na possibilidade de acompanhar o desenvolvimento acadêmico do aluno em termos éticos-profissionais e verificar que a formação serviu como um importante meio de transformação social e pessoal. Os docentes se sentem recompensados ao serem parte integrante do processo de construção de um futuro profissional.

*É muito gratificante pegar o aluno em fase inicial e acompanhar lá na frente o crescimento na formação desse futuro profissional. Eu vejo mudanças tanto na parte pessoal quanto social de cidadania. É prazeroso enxergar essa transformação.* (E6 - docente/biologia)

*O lado bom é quando você consegue acompanhar o crescimento acadêmico e o desenvolvimento da ética profissional do seu aluno. Eu percebo que, nesses anos, eu consegui transmitir, ensinar, pelo menos parte dessa minha experiência aos meus alunos.* (E3 - docente/medicina)

Ademais, apontaram como situação geradora de prazer um ambiente que oferece a possibilidade de produção de conhecimento, que pode ser alcançado por meio de pesquisas e publicação de artigos.

*Poder construir conhecimento, eternizar algum tipo de conhecimento através do material produzido, seja publicando artigo, seja apresentando em algum evento, seja fazendo alguma roda de conversa, ou ainda fazendo alguma discussão, também é gerador de prazer.* (E1 - docente/odontologia)

*Quando a gente tem sucesso com o trabalho e consegue a publicação é muito bom, como, por exemplo, foi o lançamento de um livro e sua repercussão fora do Rio de Janeiro. Então, concluir isso é muito gratificante, porque é a premiação depois de um esforço tão grande em termos de energia.* (E9 - docente/nutrição)

Apreendeu-se que as circunstâncias que promovem o prazer no trabalho têm um impacto positivo na saúde, pois aumentam os sentimentos de satisfação, de pertencimento e de reconhecimento, além de fortalecer a identidade profissional e a subjetividade.

*O meu trabalho me transforma, e na medida em que ele me transforma, me torna mais forte, reforça a minha identidade, e isso tem uma repercussão positiva no meu corpo. Então, acho que nesse sentido o trabalho me reconstrói e me traz coisas boas no ponto de vista da saúde.* (E24 - docente/enfermagem)

*O trabalho é positivo para minha saúde, porque o profissional que gosta da profissão, aquilo tem um bom retorno para ele. A minha saúde é boa por exatamente executar essas coisas que me dão prazer.* (E18 - docente/medicina)

### Situações geradoras de sofrimento e de dificuldades no cotidiano laboral

Essa segunda categoria correspondeu a 26 USs, o que equivale a 22% do total de unidades de registro, em que se apresentam os conteúdos representativos de sofrimento no e pelo trabalho. Assim, os docentes afirmaram que a falta de investimentos em ciência e tecnologia, e o corte de verbas para as universidades públicas causam sofrimento e dificultam o desenvolvimento de atividades laborais docentes, pois esses recursos são essenciais para a vida profissional, conforme evidenciado nos discursos dos participantes.

*O sofrimento está relacionado à pesquisa, à questão de captação de fomento, custo para desenvolver a pesquisa. Então, a insatisfação é não poder avançar mais, dar passos mais longos pela limitação financeira, que está bem mais difícil.* (E13 - docente/nutrição)

*As dificuldades são as condições que a gente se encontra e que vêm piorando nos últimos anos. Sua colônia de ratos não pode mais reproduzir, porque não tem mais lugar no biotério, porque não tem ração, porque o ar condicionado quebrou. O não pagamento de bolsas de auxílio de pesquisas. Tudo isso vem atrapalhando bastante.* (E15 - docente/biologia)

Os docentes salientam que o trabalho acaba por invadir o tempo de não trabalho devido à diversidade e complexidade de tarefas, e à alta demanda laboral. Isso ocorre porque não conseguem cumprir todas as atividades no horário de trabalho formal, o que os obriga a levar para casa tarefas que não conseguiram concluir no ambiente laboral. Esta situação é um exemplo da polivalência e multifuncionalidade do trabalho docente.

*Eu posso afirmar categoricamente que todos nós trabalhamos mais do que 40 horas por semana, e a gente passa a naturalizar os finais de semana como carga horária de trabalho para aquilo que a gente não dá conta durante a semana. Isso gera sofrimento, porque, quando eu vou dormir às 3 horas da manhã, foi um cinema que eu não fui, foi um tempo com o meu filho que eu não tive*. (E11 - docente/odontologia)

*Além da parte docente, a gente ainda presta trabalho assistencial, que é grande. E a demanda por esse trabalho é importante para manter o serviço funcionando, então a gente tem que ser mão de obra de trabalho assistencial também, e dá a impressão que o docente só cuida dos alunos, quando na verdade a gente tem uma carga de trabalho em relação ao paciente, em relação à administração do serviço.* (E17 - docente/medicina)

A competitividade que existe no ambiente de trabalho universitário foi fator destacado pelos participantes como situação de sofrimento. Porém, dialeticamente, foi mencionado na categoria anterior o bom relacionamento com os colegas como aspecto que configura prazer.

*Às vezes, a gente tem vontade de desistir, porque não tem como competir com grandes centros e com a competição aqui entre os docentes, porque, quando a gente tem “a farinha pouca”, os grupos que detêm o poder, eles acabam dando aquele jeito de manter esse poder, então isso gera uma competição desleal ou acirrada, e desanima.* (E27 - docente/biologia)

*Você não pode contar com seu colega de trabalho, pois ele parece se sentir ameaçado na presença de um outro. Outra coisa que tem que ser dita é que o mundo acadêmico/científico, é um mundo de divisão social do trabalho e um mundo muito competitivo.* (E29 - docente/enfermagem)

As condições de trabalho inadequadas, combinadas com a infraestrutura precária da universidade, dificultam o crescimento profissional, tanto em termos de infraestrutura física quanto em termos de recursos materiais necessários para a prática docente no ensino e/ou na assistência, as quais foram associadas à situação de sofrimento.

*As condições de trabalho são ruins e a disponibilidade de recursos físicos e estruturais é frágil, quase inexistente. O meu suporte administrativo é ruim, o equipamento também, os equipamentos de informática são obsoletos.* (E2 - docente/enfermagem)

*Tem fatores físicos de limitação, como, por exemplo, dentro da faculdade de medicina e dentro do hospital. Tem pouquíssimas áreas que você possa usar para o ensino, ou seja, juntar os alunos para discutir casos, enfim você não tem sala de aula, tem uma limitação física e de equipamentos para a docência.* (E3 - docente/medicina)

*Outra coisa muito importante é a insalubridade, pois não consideram isso aqui insalubre, mas tem o esgoto da clínica de cima: saliva, sangue, porque tem cirurgia. Geralmente, são cirurgias infectadas, para remover abscesso, essas coisas, então a gente fica respirando isso porque a gente fecha e tem ar condicionado, então você é exposto a isso*. (E7 - docente/odontologia)

## DISCUSSÃO

Os resultados apontaram diversas situações causadoras de prazer e de sofrimento vivenciadas pelos docentes universitários. Captaram-se sentimentos de satisfação e insatisfação, estímulo e desânimo, bem-estar, e inquietação, os quais não são neutros em relação à saúde dos trabalhadores, podendo, então, assegurar ou desestabilizar a saúde desses profissionais^(1)^.

Evidenciou-se que docentes satisfeitos com seu trabalho poderão contribuir para um processo de formação dinâmico, crítico-reflexivo e de alta qualidade. Os profissionais satisfeitos e bem realizados mostram que estão comprometidos com o ensino, a pesquisa e a extensão, e estão dispostos a ajudar sistematicamente os alunos a aprender. Além disso, pesquisa realizada na região Sul do Brasil enfatizou que docentes felizes têm a capacidade de criar novos conhecimentos, compartilhar conhecimentos, integrar ensino com assistência e serviço, e formar cidadãos mais preparados para a realidade dinâmica da sociedade moderna^(14)^.

Assim, a integração entre ensino-pesquisa-extensão aumenta o trabalho acadêmico, aproxima a universidade da sociedade, e aumenta o senso crítico. Também destaca o lado social da prática acadêmica^(15)^. No que se refere ao processo de relacionamento interpessoal entre indivíduos, a comunicação possibilita a partilha de conhecimento, além de sentimentos, emoções e pontos de vista. O bom relacionamento interpessoal fomenta a interação entre as pessoas e intensifica os vínculos no local de trabalho, tornando o ambiente mais agradável e propício para realização das tarefas. Com a rede de amizades, a pessoa se sente segura e acolhida, desenvolvendo habilidades para superar obstáculos, o que pode estender sua permanência no emprego^(16)^.

Nessa perspectiva, as interações sociais entre docentes e discentes possibilitam a criação de laços de cordialidade e/ou amizade no ambiente de trabalho, e são benéficas tanto para a saúde dos trabalhadores quanto para a estrutura organizacional do trabalho. Assim, quando as relações laborais são benéficas, mostrando respeito, escuta, interação e cooperação, o prazer predomina e a individualidade dos trabalhadores é respeitada, além da saúde e do bem-estar do grupo profissional^(17)^.

A Psicodinâmica do Trabalho enfatiza que a capacidade de mudança, criatividade, colaboração, confiança e percepção de utilidade social são elementos cruciais para sensação de prazer no trabalho e no trabalho em si^(18)^.

Na temática sobre afinidade por uma área específica e troca de pensamentos e reflexões por meio de debates e pesquisas científicas, sabe-se que auxilia no processo de socialização entre as pessoas. Essa interação entre professor e aluno é extremamente benéfica tanto para o professor, que tem a chance de se sentir ouvido e apreciado em sua área de estudo, quanto para o aluno, que pode adquirir conhecimentos de alguém com ampla experiência no assunto^(19)^.

Em revisão sistemática, destacou-se que a carreira acadêmica tem possibilitado a conexão entre ensino e pesquisa, além de intensificar a formação de novos pesquisadores e a publicação técnica. Isso representa oportunidade de autorrealização, uma vez que se trata de uma atividade vista como elevada^(20)^.

Dessa forma, é responsabilidade do professor abordar com os alunos conteúdos ligados aos princípios da profissão e aos saberes avançados que surgem de estudos recentes. Ademais, é altamente aconselhável que o professor produza conhecimento através de pesquisas, fazendo descobertas inéditas e divulgando-as por meio da publicação de artigos científicos em periódicos de grande impacto internacional^(20)^.

Nas instituições de ensino superior, a geração de conhecimento é intrínseca ao trabalho do professor. Esses profissionais são avaliados tanto em termos quantitativos quanto em termos qualitativos, o que estimula o docente a se manter produtivo no ambiente acadêmico, levando a um maior destaque na carreira docente^(21)^.

Os resultados evidenciados na segunda categoria destacam que, com a globalização, as tendências econômicas e a competitividade no trabalho docente provocaram mudanças na organização do trabalho, demandando um rendimento profissional acelerado. Isso resulta em transformações nas empresas públicas e privadas, que priorizam o uso de alta tecnologia, nanotecnologia, inteligência artificial, entre outros. Esse contexto demanda uma rápida adaptação, impactando diretamente a saúde física e mental dos docentes. Desse modo, tem provocado um crescimento dos índices de estresse nos trabalhadores, e o trabalho do docente universitário não é imune a essa situação^(22)^.

A falta de investimento no setor público de educação de nível superior ficou evidente nas falas dos participantes, pois a manutenção e investimentos em equipamentos básicos ao labor, como computadores, aparelho de refrigeração do ar ambiente, entre outros, não ocorrem de maneira adequada, o que causa sofrimento. Associado a isso, tem o corte de verbas, que dificulta o trabalho docente, e sem o incentivo financeiro, muitos projetos de pesquisa são inviabilizados^(23)^.

A sobrecarga e o acúmulo de tarefas levam os docentes a se adaptar a um ritmo acelerado de trabalho. Com isso, têm dificuldade para se desligar mentalmente das responsabilidades e sentem culpa ao descansar, pensando nas tarefas pendentes. Muitos se frustram por só se sentirem úteis quando estão trabalhando intensamente^(24,25)^.

Esse resultado se assemelha ao de estudo conduzido com docentes universitários de uma instituição pública situada em todas as regiões do Brasil, que revelou insatisfação com a estrutura do trabalho devido à pressão para cumprir prazos, ao aumento do tempo dedicado ao trabalho, à cobrança por resultados e ao aumento da carga horária dos docentes^(26-28)^.

Desse modo, os docentes acabam executando tarefas no ambiente doméstico, em seu lar, incluindo a orientação de pesquisas e trabalhos de conclusão, a redação de artigos científicos, o preenchimento de formulários para as entidades financiadoras, a execução de avaliações para periódicos científicos, entre outras tarefas^(19)^.

É importante destacar que essa invasão do espaço pessoal pelo ambiente corporativo pode afetar negativamente o professor, não apenas por ter que sacrificar seu tempo de descanso e lazer para cumprir suas obrigações laborais, mas também porque esse profissional precisa usar seus próprios recursos materiais, como computador, internet, energia elétrica, entre outros, afetando também seus gastos financeiros^(29)^.

Nesse sentido, a sobrecarga relacionada às necessidades e aos excessos de trabalho presentes na pesquisa, ensino e extensão resultantes do acúmulo de trabalho entre os docentes universitários, considerando a desproporção na relação entre alunos e docentes, bem como a variedade de tarefas na graduação, pós-graduação, pesquisa e extensão, é prejudicial ao bem-estar dos docentes. Essas atividades, juntamente com as tarefas administrativas e burocráticas, são intensificadas pela escassez de pessoal técnico-administrativo^(27)^.

Essa circunstância está relacionada à alta pressão por produtividade imposta por metas, uma característica marcante do contexto neoliberal. Nesse cenário, destaca-se a carga excessiva de trabalho do professor universitário. A atividade docente passa a ser marcada pelo enfraquecimento dos vínculos interpessoais e das relações profissionais, além do acúmulo de funções que intensificam o ritmo de trabalho nas instituições de ensino. Isso configura uma nova estrutura de trabalho na educação, que favorece métodos de avaliação individual, estimula a competição e se afasta da ciência como prática coletiva, priorizando a quantidade de publicações acadêmicas^(25)^.

Esses resultados apontam para as particularidades e demandas do processo de trabalho do professor de mestrado e doutorado ligadas ao *workaholism.* Assim, os *workaholi*cs trabalham de maneira excessiva e compulsiva, sacrificando momentos de lazer ou interação social e familiar em prol da intensidade do trabalho. No entanto, frequentemente, não alcançam o rendimento esperado, pois o *workaholism* eleva a probabilidade de incapacidade laboral devido à deterioração da saúde biopsicossocial^(30)^.

Em relação à competitividade e vaidade, são características intrínsecas ao ambiente acadêmico, pois nele se encontram indivíduos de alto nível social, detentores e disseminadores de conhecimento científico avançado. No entanto, ressalta-se que, com a implementação do modelo neoliberal, essa circunstância se intensificou^(31)^.

Salienta-se também que a apropriação da subjetividade do trabalhador está vinculada às características do capitalismo neoliberal, em que a subjetividade dos trabalhadores é severamente impactada pelo fomento e estímulo à competição e ao individualismo, gerados pela reestruturação das relações de trabalho^(32)^.

Evidências indicam que o neoliberalismo emprega táticas que ampliam o controle sobre a subjetividade do trabalhador, visando fomentar uma competição feroz e o individualismo. Portanto, aumenta-se a desunião entre os trabalhadores e sua habilidade de organização, resultando em um ambiente de trabalho hostil em termos de relações interpessoais^(33)^.

Cabe destacar que, no início do século XXI, com o crescimento acelerado das instituições de ensino superior no Brasil, a Coordenação de Aperfeiçoamento de Pessoal de Nível Superior (CAPES) começou a implementar alguns critérios de avaliação para categorizar essas instituições nas áreas de pesquisa, ensino e extensão. Os critérios fundamentais de avaliação incluem o regime de trabalho da maioria dos docentes, a competência dos docentes para pesquisar, a estrutura de produção e disseminação de conhecimento científico, além da pós-graduação *stricto sensu*. Em outras palavras, para uma instituição obter uma boa classificação na CAPES, ela precisa de um corpo docente altamente qualificado, totalmente dedicado à universidade, empenhado em produzir e disseminar pesquisas de alta qualidade e quantidade, o que resulta na captação de recursos para a instituição e para os docentes^(27)^.

Dessa forma, além das responsabilidades inerentes à profissão de professor, o ambiente de trabalho docente tem promovido uma busca incessante por produtividade, evidenciada por publicações resultantes de estudos em revistas de renome internacional. Portanto, os órgãos de incentivo à pesquisa e à universidade incentivam os docentes a produzir artigos científicos em larga escala, o que resulta em maior reconhecimento na carreira, maior visibilidade para a universidade, e benefícios financeiros, devido às bolsas de pesquisa concedidas aos docentes, à progressão na carreira e ao financiamento da instituição para realização de mais pesquisas^(34)^.

A infraestrutura inadequada também foi abordada pelos docentes, resultado da deterioração das condições de trabalho, marcada por espaços mal planejados, falta de salas para os trabalhadores, iluminação insuficiente e/ou inadequada, entre outros aspectos, que tem sido apontada como um obstáculo para o avanço do trabalho nos setores públicos.

Desse modo, frequentemente, a infraestrutura física das instituições públicas educacionais e de saúde é inadequada, apresentando uma série de distorções como: salas de tamanhos inadequados para o público-alvo; corredores estreitos; ausência de rampas; má iluminação; ventilação ou climatização insuficientes; instalações físicas antigas e em estado de deterioração; banheiros insuficientes para a quantidade de trabalhadores; escassez de recursos materiais; manutenção deficiente dos recursos já existentes; e até mesmo recursos obsoletos^(27)^.

Essa escassez e a inadequação das condições laborais causam profundo sofrimento psicológico, adoecimento e, até mesmo, um aumento na taxa de abandono da carreira. Esse ambiente de trabalho apresenta desafios para o trabalhador no desempenho de suas funções e, sem dúvida, as tarefas realizadas ficam aquém do necessário^(30)^.

Cabe asseverar que o sofrimento é visto como uma experiência subjetiva intermediária entre uma doença mental descontrolada e o conforto (ou bem-estar) psicológico. Portanto, o debate não termina no sofrimento em si, mas na compreensão de que ele pode surgir como uma condição patológica. Nessa perspectiva, o sofrimento patológico surge quando todas as oportunidades de adaptação ou ajuste à organização do trabalho pelo indivíduo foram esgotadas, e a conexão subjetiva com essa organização do trabalho está impedida^(35)^.

Dessa forma, quando a reorganização do trabalho se torna inviável, quando a relação entre o trabalhador e a organização se torna muito desvinculada, ou seja, se as demandas da organização ou as condições de trabalho forem extremamente adversas, o trabalhador não consegue estabelecer um processo saudável de adaptação por meio de qualquer tipo de mediação, resultando em sofrimento patológico e doença^(36)^.

### Limitações do estudo

A limitação deste estudo está em ter sido desenvolvido em um único cenário, o que pode comprometer a generalização dos resultados. Assim, sugere-se o desenvolvimento de pesquisas em outros cenários públicos e privados, e demais áreas de conhecimento como as ciências humanas, exatas, avaliando-se as especificidades de outras áreas distintas à área da saúde.

### Contribuições para a área da enfermagem

O estudo contribui de forma significativa no campo da saúde do trabalhador e formação, reforçando a necessidade de equilíbrio entre as situações geradoras de prazer e sofrimento no ensino universitário em saúde. Essas contribuições podem orientar ações institucionais, políticas educacionais e práticas pedagógicas comprometidas com a saúde dos docentes e com condições de trabalho favoráveis para a excelência do ensino na área de saúde nas universidades públicas brasileiras.

## CONSIDERAÇÕES FINAIS

Em relação à visão dos docentes sobre as circunstâncias geradoras de prazer e sofrimento no trabalho, constatou-se que as circunstâncias que proporcionam prazer estão ligadas: ao progresso das atividades de ensino, pesquisa e extensão; às interações interpessoais com os alunos e colegas de trabalho; à relevância da formação como meio de mobilização e mudanças pessoais e sociais; e à produção de conhecimento materializado em livros, artigos e trabalhos científicos.

As circunstâncias identificadas como causadoras de sofrimento e desafios no trabalho docente foram: a escassez de investimentos públicos na educação e, consequentemente, na universidade; a sobrecarga de atividades devido ao grande volume de trabalho; a competição presente no âmbito universitário; a infraestrutura insuficiente; e as condições laborais inadequadas.

## Data Availability

Os dados de pesquisa estão disponíveis no corpo do artigo.
